# A phase II trial of gefitinib with 5-fluorouracil, leucovorin, and irinotecan in patients with colorectal cancer

**DOI:** 10.1038/sj.bjc.6602569

**Published:** 2005-05-03

**Authors:** M L Veronese, W Sun, B Giantonio, J Berlin, J Shults, L Davis, D G Haller, P J O'Dwyer

**Affiliations:** 1 Abramson Cancer Center at the University of Pennsylvania, Philadelphia, PA 19104, USA

**Keywords:** colon cancer, gefitinib, EGFR, irinotecan

## Abstract

Inhibition of epidermal growth factor receptor (EGFR) signalling contributes to the therapy of colorectal cancer. Gefitinib, an oral EGFR tyrosine kinase inhibitor, shows supra-additive growth inhibition with irinotecan and fluoropyrimidines in xenograft models. We designed a study to determine the tolerability and efficacy of gefitinib in combination with irinotecan, infusional 5-fluorouracil (5-FU) and leucovorin (LV), on a 2-week schedule. Among 13 patients with advanced colorectal cancer, 10 required dose reductions of irinotecan and 5-FU because of dehydration, diarrhoea, and neutropenia, seven of whom required hospitalisation, three with neutropenic fever. One patient achieved partial response and seven had disease stabilisation. The combination of this standard chemotherapy regimen with gefitinib is associated with excessive toxicity, suggesting an interaction at a pharmacokinetic or pharmacodynamic level.

The addition of irinotecan to 5-fluorouracil (5-FU) results in response rates (RR) of 30–40%, and improved survival to about 14–16 months in metastatic colorectal cancer (Douillard *et al*, 2000; Saltz *et al*, 2000). Weekly and every 3-week schedules of irinotecan demonstrate comparable toxicity and efficacy (Rougier *et al*, 1997; Fusch *et al*, 2003), but the weekly combination of irinotecan/5-FU shows excessive toxicity (Rothenberg *et al*, 2001). The FOLFIRI regimen (Douillard *et al*, 2000) is less toxic, and infusional 5-FU or capecitabine combinations are favoured (Han *et al*, 2003; Tewes *et al*, 2003; Bajetta *et al*, 2004; Jordan *et al*, 2004).

Expression of the epidermal growth factor receptor (EGFR) has been associated with outcome in colon cancer (Mayer *et al*, 1993; Saloman *et al*, 1995). Gefitinib is an oral EGFR tyrosine kinase inhibitor with an excellent safety profile. The combination of gefitinib and irinotecan has shown supra-additive growth inhibition in colorectal cancer cell lines and in xenograft models (Koizumi *et al*, 2004). In a human head and neck cancer cell line treated with gefitinib before or during 5′-deoxy-5-fluorouridine (5′-dFUR) treatment, there was a strong synergistic cytotoxic activity, associated with altered target enzyme expression (Magne *et al*, 2003). Clinical studies by Saltz and by Cunningham demonstrated the therapeutic role of an antibody directed to EGFR (Cunningham *et al*, 2004; Saltz *et al*, 2004).

We conducted a phase II study to determine the tolerability and response with gefitinib together with irinotecan, infusional 5-FU, and LV. The coadministration of these agents was associated with unexpectedly severe gastrointestinal toxicity and myelosuppression, and the RR in this small cohort was modest at best.

## PATIENTS AND METHODS

### Eligibility

Eligible patients were over 18 years with advanced or recurrent colorectal adenocarcinoma. No prior chemotherapy other than adjuvant 5-FU was allowed. Patients were required to have measurable disease. Eligibility criteria also included performance status 0–2 (ECOG); adequate bone marrow (neutrophils ⩾1500 mm^−3^, platelets ⩾100 000 mm^−3^), renal (creatinine ⩽1.5 times the upper limit of normal (ULN)), and hepatic (bilirubin <1.5 times ULN, and AST/ALT ⩽2 times ULN (<5 × ULN if liver metastasis) function. The study was approved by the University of Pennsylvania institutional review board. All patients signed written informed consent.

### Pretreatment evaluation and follow-up

Pretreatment evaluation consisted of a history and physical examination, full blood count, electrolytes, creatinine, liver function tests and carcinoembryonic antigen (CEA), urinalysis, electrocardiogram, and baseline imaging. Clinical, haematologic, and biochemical evaluations were performed every 2 weeks. Toxicity was graded according to the National Cancer Institute Common Toxicity Criteria (NCI-CTC).

### Study design and treatment administration

This study was a nonrandomised safety and efficacy trial of 5-FU, LV, and irinotecan in combination with gefitinib in patients with advanced colorectal cancer. Patients received LV 400 mg m^−2^ as a 2 h intravenous (i.v.) infusion, followed immediately by bolus 5-FU 400 mg m^−2^ and a 22 h i.v. infusion of 5-FU 600 mg m^−2^, on days 1 and 2, and irinotecan 180 mg m^−2^ as a 90 min i.v. infusion during LV on day 1 only (5-FU400–600/I180). Treatment was repeated every 2 weeks and one cycle consisted of 28 days. Gefitinib 250 mg orally was administered continuously, beginning on day 1 (G250). Antiemetic prophylaxis with a 5-hydroxytryptamine-3-receptor antagonist and dexamethasone was used. Dose modifications were made for myelosuppression (5-FU and irinotecan), diarrhoea (5-FU, irinotecan, and gefitinib), mucositis (5-FU only), and skin rash (gefitinib only). The LV dose was not modified.

### Response evaluation

Measurable lesions were reassessed according to the Response Evaluation Criteria in Solid Tumors (RECIST) (Therasse *et al*, 2000) after cycles 2 and 4, and then every three cycles until progression.

### Statistical analysis

The purpose of this study was to assess the safety and efficacy of gefitinib, irinotecan, LV, and 5-FU in combination. We wished to be able to discern a toxicity rate of 29% with the new regimen. With an accrual of 50 patients, we would have 80% power to distinguish that level of toxicity from a 12% rate observed in prior studies (Douillard *et al*, 2000). We targeted an RR of 60%, and planned to accrue 50 patients to give a 95% confidence interval of 46–72%. All analyses were conducted using Stata 8.0, with two-sided tests of hypotheses and a *P*<0.05 criterion for statistical significance.

## RESULTS

### Patient characteristics

The demographic characteristics of the 13 patients are shown (Table 1). All were evaluable for toxicity and 12 for response. One patient was removed from study because of excessive first cycle toxicity.

### Treatment administration

A total of 10 patients required dose reductions of irinotecan and 5-FU (Table 2). The first seven patients received I180/5-FU400–600/G250. Four experienced grade 3 neutropenia and/or dehydration during the first cycle of therapy and required 20% dose reduction of irinotecan and 5-FU (I150/5-FU320–600/G250). Of these seven, four required an additional 20% dose reduction (I120/5-FU200–500/G250) because of dehydration or neutropenia. Because of this unacceptable level of toxicity, subsequent patients were treated at a reduced dose of irinotecan and 5-FU (I150/5-FU320–600/G250). Of four patients treated at these doses, three developed grade 4 neutropenia after the first cycle and two grade 3 diarrhoea, requiring an additional 20% dose reduction (I120/5-FU200–500). For safety reasons, these doses were used in the subsequent patients. This dose level was well tolerated by eight patients in total (two new, six reduced) (Table 2). No dose changes were made for gefitinib.

### Toxicity

The most frequent grade 3/4 adverse events during this study were gastrointestinal (mostly diarrhoea, 54%) and haematologic (neutropenia, 62%) (Table 3). Seven patients (54%) developed grade 3 or 4 dehydration from diarrhoea, and all required hospitalisation. Two were removed from study because of toxicity.

Eight patients (62%) developed grade 3 or 4 neutropenia, of whom three experienced neutropenic fever. One expired from sepsis after receiving one cycle of therapy. Of these eight patients, four had concomitant grade 3/4 diarrhoea.

Acneiform skin rash occurred in eight patients, and was mild and reversible. Grade 1/2 nausea and vomiting occurred in seven patients (54%). Mucositis and fatigue were generally mild.

### Response

The primary efficacy end point was RR. One patient achieved partial response (PR) for seven cycles (8%, 95% CI 0–38%). Seven of the 12 (58%, 95% CI 28–85%) evaluable patients had disease stabilisation that persisted for more than six cycles in six. Based on the excessive toxicity and low level of activity, after review of all results, the trial was closed.

## DISCUSSION

The combination of gefitinib with irinotecan, 5-FU, and LV demonstrated excessive gastrointestinal and haematologic toxicity in over half of the patients, despite the use of an infusional 5-FU schedule. The tolerable doses of irinotecan and 5-FU with gefitinib were one-third less than those commonly used. With these doses, activity appeared no better than expected, which prompted early closure of the study.

The excessive toxicity suggests an interaction between gefitinib and the chemotherapy. An interaction between gefitinib and 5-FU seems unlikely. Full doses of 5-FU/LV with gefitinib were tolerated on various schedules, without apparent pharmacokinetic interactions (Hammond *et al*, 2001). Gefitinib 250 mg can be administered with full doses of oxaliplatin/5-FU with tolerable toxicity, although a higher incidence of diarrhoea may suggest a possible pharmacodynamic interaction (Fisher *et al*, 2004).

The profile of side effects in our study suggests an interaction with irinotecan rather than with 5-FU. Irinotecan is metabolised in the liver by several enzyme systems (Kawato *et al*, 1991; Rivory and Robert, 1995; Haaz *et al*, 1997; Xie *et al*, 2002). Cleavage by carboxylesterase yields the active metabolite SN-38, which is inactivated by glucuronide conjugation by uridine diphosphate glucuronosyltransferase 1A1 (UGT1A1) (Haaz *et al*, 1997), variable activity of which is a determinant of toxicity (Innocenti *et al*, 2004). Another pathway involves oxidation of the terminal piperidine ring by the P450 enzyme, CYP3A4 (Rivory *et al*, 1996; Dodds *et al*, 1998), of which gefitinib is a weak inhibitor. Additionally, mild elevations of bilirubin have been shown to predict neutropenia with weekly irinotecan, implicating hepatic excretion (Meyerhardt *et al*, 2004).

Iacono *et al* reported a study of gefitinib and irinotecan in children. Concomitant administration of gefitinib reduced irinotecan clearance and increased the AUC of SN-38 (Iacono *et al*, 2004). In another phase I clinical trial (Chau *et al*, 2004) of gefitinib with irinotecan, dose reduction was required. We propose that the interaction of irinotecan and gefitinib in our trial is likely to have been pharmacokinetic, resulting in greater than anticipated exposure to irinotecan and its active metabolite. Interestingly, Messersmith *et al* (2004) have recently reported excessive toxicity requiring early closure of the study in a phase I trial of a combination of erlotinib with reduced doses of irinotecan and infusional 5-FU. A pharmacokinetic interaction between irinotecan, 5-FU, and erlotinib could not explain the toxicity. Additionally, blockage of the EGFR may not account for this level of toxicity since combination of Erbitux with irinotecan has not resulted in such severe toxicity (Cunningham *et al*, 2004). An interaction between gefitinib and the multidrug resistance ABC transporter could have contributed to toxicity. Gefitinib inhibits ABCG2-dependent active drug extrusion, which mediates irinotecan efflux and protects cells from irinotecan toxicity (Wierdl *et al*, 2003; Ozvegy-Laczka *et al*, 2004).

While both of these possibilities need consideration in using these drugs in combination, the implied increase in sources of intrapatient variability was perceived as a safety hazard for further development on a solely empirical basis.

## Figures and Tables

**Table 1:** Patient characteristics

No. of patients	
Entered	13
Assessable	12
	
Sex	
Male	6
Female	7
	
Age (years)	
Range	49–72
Median	58
	
ECOG performance status	
1	0
1	2
2	1
	
Site of tumour	
Colon	12
Rectum	1
Liver involvement	10

**Table 2:** Toxicity related to the combination of irinotecan/5-FU/gefitinib by dose

	***N***		**Maximum toxicity/grade**							
	**New^a^**	**Total^b^**	**Nonhaematologic**				**Haematologic**			
**Dose**			**G 1**	**G 2**	**G 3**	**G 4**	**G 1**	**G 2**	**G 3**	**G 4**
I180/5-FU400–600	7	7	7	7	4	1	2	4	3	2
I150/5-FU320–500	4	7	4	4	3	0	0	1	1	2
I120/5-FU200–500	2	8	1	2	0	0	0	0	0	0

Gefitinib dose was 250 mg daily and it was not reduced. 5-FU=5-fluorouracil.

a Number of patients who received the given irinotecan and 5-FU doses on cycle 1.

b Number of patients who received the given irinotecan and 5-FU doses on cycle 1 plus as reductions in subsequent cycles.

**Table 3:** Toxicity related to the combination of irinotecan/FU/LV/gefitinib by cycle

**Toxicity**	**Cycle 1 (*N*=13)**	**Cycle 2 (*N*=10)**	**Cycle 3 (*N*=7)**	**Cycle 4 (*N*=7)**	**Cycle 5 (*N*=6)**	**Cycle 6 (*N*=4)**
Diarrhoea						
Grade 1 or 2	5	3	2	2	3	2
Grade 3 or 4	2	2	0	2	0	1
						
Nausea/vomiting						
Grade 1 or 2	7	5	4	2	2	3
Grade 3 or 4	0	0	0	0	0	0
						
Dehydration						
Grade 1 or 2	1	0	0	0	0	0
Grade 3 or 4	3	3	0	1	0	0
						
Mucositis						
Grade 1 or 2	2	0	0	0	0	0
Grade 3 or 4	0	0	0	0	0	0
						
Fatigue						
Grade 1 or 2	6	5	1	1	2	2
Grade 3 or 4	0	0	0	0	0	0
						
Skin rash						
Grade 1 or 2	6	3	3	1	1	1
Grade 3 or 4	0	0	0	0	0	0
						
Neutropenia						
Grade 1 or 2	2	1	1	1	1	0
Grade 3 or 4	5	1	0	0	2	0
						
Neutropenic fever	2	1	0	0	0	0
						
Anaemia						
Grade 1 or 2	5	0	1	0	0	
Grade 3 or 4	1	0	0	0	0	

5-FU=5-fluorouracil; LV=leucovorin.
